# Relationships of Late Pleistocene giant deer as revealed by *Sinomegaceros* mitogenomes from East Asia

**DOI:** 10.1016/j.isci.2023.108406

**Published:** 2023-11-07

**Authors:** Bo Xiao, Alba Rey-lglesia, Junxia Yuan, Jiaming Hu, Shiwen Song, Yamei Hou, Xi Chen, Mietje Germonpré, Lei Bao, Siren Wang, Taogetongqimuge, Lbova Liudmila Valentinovna, Adrian M. Lister, Xulong Lai, Guilian Sheng

**Affiliations:** 1 State Key Laboratory of Biogeology and Environmental Geology, China University of Geosciences, Wuhan 430078, China; 2 School of Earth Sciences, China University of Geosciences, Wuhan 430074, China; 3 Globe Institute, University of Copenhagen, Copenhagen, 1350 Copenhagen K, Denmark; 4 Faculty of Materials Science and Chemistry, China University of Geosciences, Wuhan 430078, China; 5 School of Environmental Studies, China University of Geosciences, Wuhan 430078, China; 6 Key Laboratory of Vertebrate Evolution and Human Origins, Institute of Vertebrate Paleontology and Paleoanthropology, Chinese Academy of Sciences, Beijing 100044, China; 7 Department of Cultural Heritage and Museology, Nanjing Normal University, Nanjing 210046, China; 8 Royal Belgian Institute of Natural Sciences, 1000 Brussels, Belgium; 9 Ordos Institute of Cultural Relics and Archaeology, Ordos 017010, China; 10 Daqing Museum, Daqing 163319, China; 11 Wushen Banner Museum, Ordos 017399, China; 12 Graduate School of International Relations, Peter the Great St. Petersburg Polytechnic University, St. Petersburg, Grazhdansky Av., 28, Russia; 13 Natural History Museum, London SW7 5BD, UK

**Keywords:** Evolutionary biology, Paleobiology, Paleogenetics

## Abstract

The giant deer, widespread in northern Eurasia during the Late Pleistocene, have been classified as western *Megaloceros* and eastern *Sinomegaceros* through morphological studies. While *Megaloceros*’s evolutionary history has been unveiled through mitogenomes, *Sinomegaceros* remains molecularly unexplored. Herein, we generated mitogenomes of giant deer from East Asia. We find that, in contrast to the morphological differences between *Megaloceros* and *Sinomegaceros*, they are mixed in the mitochondrial phylogeny, and Siberian specimens suggest a range contact or overlap between these two groups. Meanwhile, one deep divergent clade and another surviving until 20.1 thousand years ago (ka) were detected in northeastern China, the latter implying this area as a potential refugium during the Last Glacial Maximum (LGM). Moreover, stable isotope analyses indicate correlations between climate-introduced vegetation changes and giant deer extinction. Our study demonstrates the genetic relationship between eastern and western giant deer and explores the promoters of their extirpation in northern East Asia.

## Highlights

• First molecular investigation of giant deer from East Asia
• Eastern and western giant deer species have an interlaced maternal relationship
• Northeastern China could be an LGM refugium for giant deer
• The distribution of Eastern giant deer extended into the Baikal region of Russia

## Graphical abstract

**Figure:**
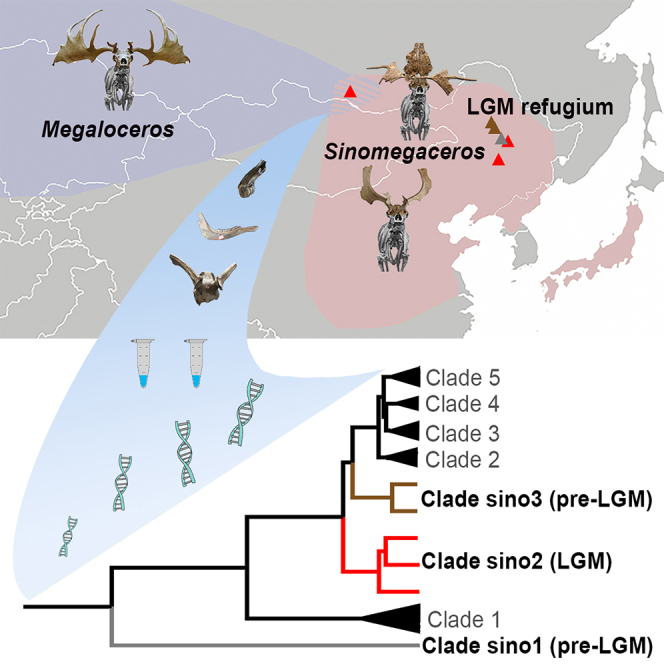

## Introduction

The giant deer or megacerines (tribe Megacerini, family Cervidae) were among the emblematic mammals distributed widely in northern Eurasia in the Pleistocene. They became extinct in the Holocene under the dramatic effects of late Quaternary climate fluctuations.^1^^,^^2^ Megacerines have been suggested to be a model group for paleoecological studies,^3^ yet their evolutionary history is still insufficiently explored.

**Figure 1:**
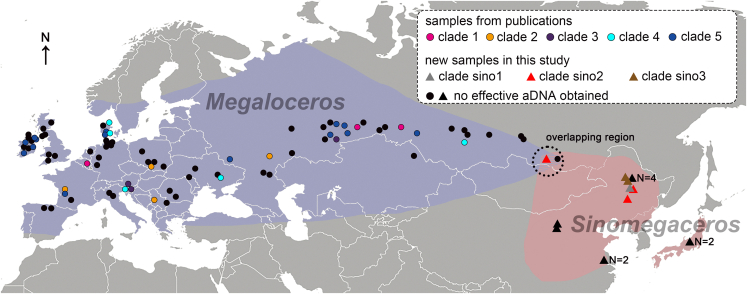
The geographical locations of megacerine individuals Light purple and pink shaded areas stand for the historical ranges of *Megaloceros giganteus* and *Sinomegaceros* spp., respectively, the latter now extending into southern Siberia based on our data. The possible overlapping region indicated in this study is circled by black dotted line. The triangles represent our samples in this study, while the circles represent samples from previous publications.^2^^,^^21^ The number of multiple samples found in the same location is numerically marked (N). The colored circles and triangles indicate samples that yielded aDNA. The black circles and triangles represent samples that did not yield aDNA. Pink, orange, purple, cyan, and blue circles stand for *Megaloceros* clades 1–5, while gray, red, and brown triangles stand for *Sinomegaceros* clades sino1–3, respectively.

**Figure 2:**
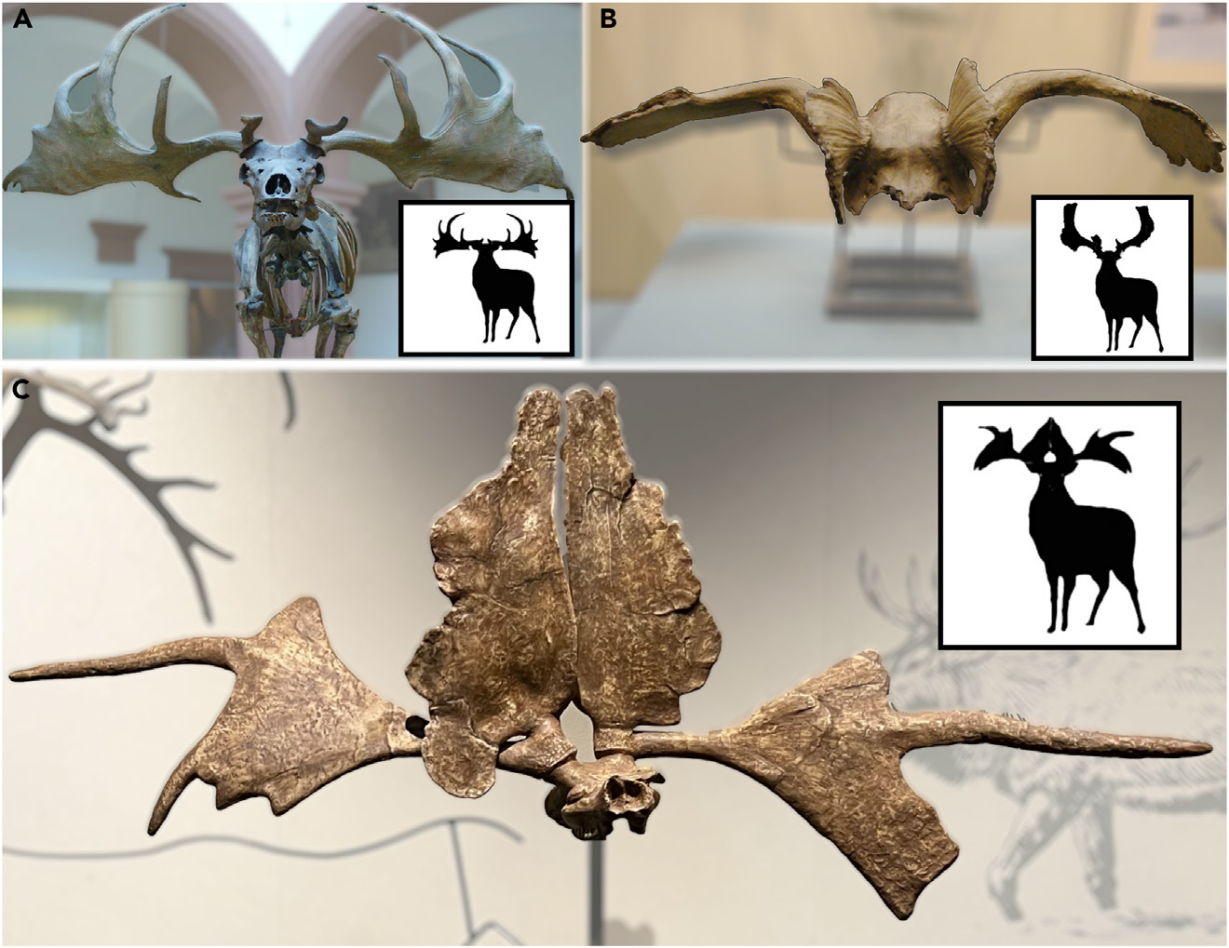
Comparison of three typical giant deer antler types in Eurasia (A) The skeleton of *M. giganteus*. Photo courtesy of Franco Atirador (website: https://commons.wikimedia.org/wiki/File:Irish_Elk_front.jpg). (B) The skull and partial antlers of *S. ordosianus* preserved in Tianjin Natural History Museum. Photo courtesy of Yu Chen. (C) The skull and antlers of *S. pachyosteus* preserved in Daqing Museum. Photo Bo Xiao.

The first appearance of undoubted megacerines in western Europe is from the Early Pleistocene (1.2 million years ago, Ma),^4^^,^^5^ while the proposed appearance for Central Eurasia was much earlier (Late Pliocene).^3^ In contrast to the controversial status of giant deer taxonomy in pre-Pleistocene, and even into the Early and Middle Pleistocene in Europe, researchers agree on a morphology-based division between the Late Pleistocene *Megaloceros giganteus* of western Eurasia and an array of Pleistocene *Sinomegaceros* species from eastern Eurasia (Figure 1).^3^^,^^6^ This is based, among other features, on the extraordinarily broad and flattened brow (basal) tine, expanded in most *Sinomegaceros* species beyond the more modest flattening seen in *Megaloceros* and unique among living and fossil deer (Figure 2). Up to nine species of *Sinomegaceros* have been proposed, from Central Asia, China, and Japan^6^ (five were recognized by Vislobokova^3^).

In the western Eurasia group, *M. giganteus*, one of the largest known species in the subfamily Cervidae, has been a focus of previous studies. The species distribution extended from Ireland to central Siberia, and with an evolutionary history dating from 400 to about 8 thousand years ago (ka).^7^^,^^8^ With regard to its phylogenetic position, rather than indicating a closer phylogenetic relationship to the living red deer (*Cervus elaphus/canadensis*),^9^ Lister et al.^5^ and Hughes et al.^10^ indicated a sister-group relationship of *M. giganteus* to the extant fallow deer (*Dama* spp.) based on both morphological and molecular evidence. The cluster of *M. giganteus* and *D. dama/mesopotamica* was confirmed by Immel et al.^11^ using two near-complete *M. giganteus* mitochondrial genomes. Recently, a decrease in the genetic diversity of this iconic species has been identified starting in Marine Isotope Stage (MIS) 3 (60–25 ka) and accelerating 22 ka during the Last Glacial Maximum (LGM) (26–19 ka), with three clades absent from the post-LGM genetic pool and two clades surviving into the Holocene.^2^ This is the first study that gave genetic insights into the population dynamics of the giant deer.

Analysis from morphological characters suggested that the eastern Eurasia cluster, *Sinomegaceros*, diverged from *Megaloceros* more than 1 Ma.^3^^,^^6^ Fossil records revealed that the range of *Sinomegaceros* covered continental areas from approximately 50° to 25°N, mainly in present-day eastern and northeastern China and the Japanese islands.^6^^,^^12^ In China, there are several well-known fossil species of *Sinomegaceros* from different stages of the Pleistocene, with *Sinomegaceros konwanlinensis*, *S. pachyosteus*, and *S. ordosianus* representing the Early, Middle, and Late Pleistocene, respectively.^13^ In Japan, *Sinomegaceros yabei* appeared from the second part of the Middle Pleistocene to the Pleistocene/Holocene boundary.^6^^,^^14^

In addition to the phylogenetic status of the giant deer, their extinction is also a focus of research. Unlike red deer (*Cervus elaphus/hanglu/canadensis*),^15^^,^^16^ moose (*Alces alces*),^17^^,^^18^ and other extant megafauna species,^19^^,^^20^ giant deer went extinct during the Holocene.^21^ There are various speculations about their disappearance. Using radiocarbon dating, stable isotopes, and archaeological evidence, researchers suggest that climate fluctuations, vegetation shifts, food competition, and human activities all contributed to the population dynamics and extinction of giant deer.^2^^,^^11^^,^^21^^,^^22^^,^^23^^,^^24^ The most recent, mid-Holocene population of *M. giganteus* spanned from eastern Europe to western Siberia.^21^ However, there is a lack of similar evidence for *Sinomegaceros* species.

Molecular studies have significant potential to illuminate the evolution and extinction of various species. Previous molecular studies have demonstrated that fossils from the Far East can provide valuable information for understanding the evolutionary history of Quaternary mammals, e.g., the cave hyena (*Crocuta* spp.),^25^ horse (*Equus dalianensis* and *E. przewalskii*),^26^ moose,^17^ and red deer.^16^ Among megacerines, all available ancient DNA studies are devoted to *Megaloceros*, the western group of giant deer, while the eastern group, *Sinomegaceros*, remains unstudied. In this study, we generate *Sinomegaceros* mitogenomic data to provide the first genetic insights into the molecular evolution of the giant deer in East Asia and their relationship to western giant deer, and bring hypothesis to elucidate their regional extirpation in northeastern China.

## Results

### Radiocarbon dating and ancient mitogenomes of giant deer in East Asia

Eight out of 16 *Sinomegaceros* specimens (Figure S1) were dated, ranging from 20,366 to 19,835 cal BP to beyond the limit of radiocarbon dating (>43,500 BP). For the specimen beyond the radiocarbon limit and the one not dated, we molecularly dated their ages to 102 ka (95% highest posterior density (HPD): 79–134 ka, CADG532) and 58 ka (95% HPD: 43–75 ka, CADG1199), respectively (Figure S2). Geographically, one specimen from Siberia, Russia (ARI38), one specimen from Japan (ARI135), and five specimens from northern and northeastern China (CADG496, CADG497, CADG532, CADG888, and CADG1199) were traced back to the pre-LGM, while two specimens from northeastern China (CADG1006 and ARI68) were dated to the LGM (Table 1 and detailed information see Table S1).

**Table 1:** Radiocarbon dates and stable isotope data of *Sinomegaceros*

Specimen	Locality	Species	Radiocarbon lab No.	Calibrated age (95% probability)/cal BP	Median age/cal BP
CADG496	Harbin, China	*S. pachyosteus*	Beta-602104	35,185–34,402	34,793
CADG497	Harbin, China	*S. pachyosteus*	Beta-560178	44,850–43,110	43,980
CADG532	Harbin, China	*S. pachyosteus*	Beta-550657	>43,500	/
CADG888	Ordos, China	*S. ordosianus*	Beta-635403	44,849–43,175	44,012
CADG1006	Harbin, China	*S. ordosianus*	Beta-643903	22,502–22,286	22,394
ARI38	Kamenka Buryatiya, Russia	*S.* cf. *ordosianus*	OxA-12116	43,133–42,424	42,778
ARI68	Yushu, China	*S. ordosianus*	OxA-36191	20,366–19,835	20,100
ARI135	Norijiko, Japan	*S. yabei*	OxA-36190	40,914–37,788	39,351

Full details are given in Table S1.

We obtained 617–3,932 unique reads for six out of 16 Late Pleistocene specimens. Six mitochondrial sequences of ancient giant deer individuals with mean coverages of 2.15- to 15.35-fold were generated. These sequences covered 83.45%–98.42% of the reference mitochondrial sequences (16,347 bp, GenBank: MW802558) (for detailed statistics see Table S2).

### Phylogenetic analyses of mitogenomes

**Figure 3:**
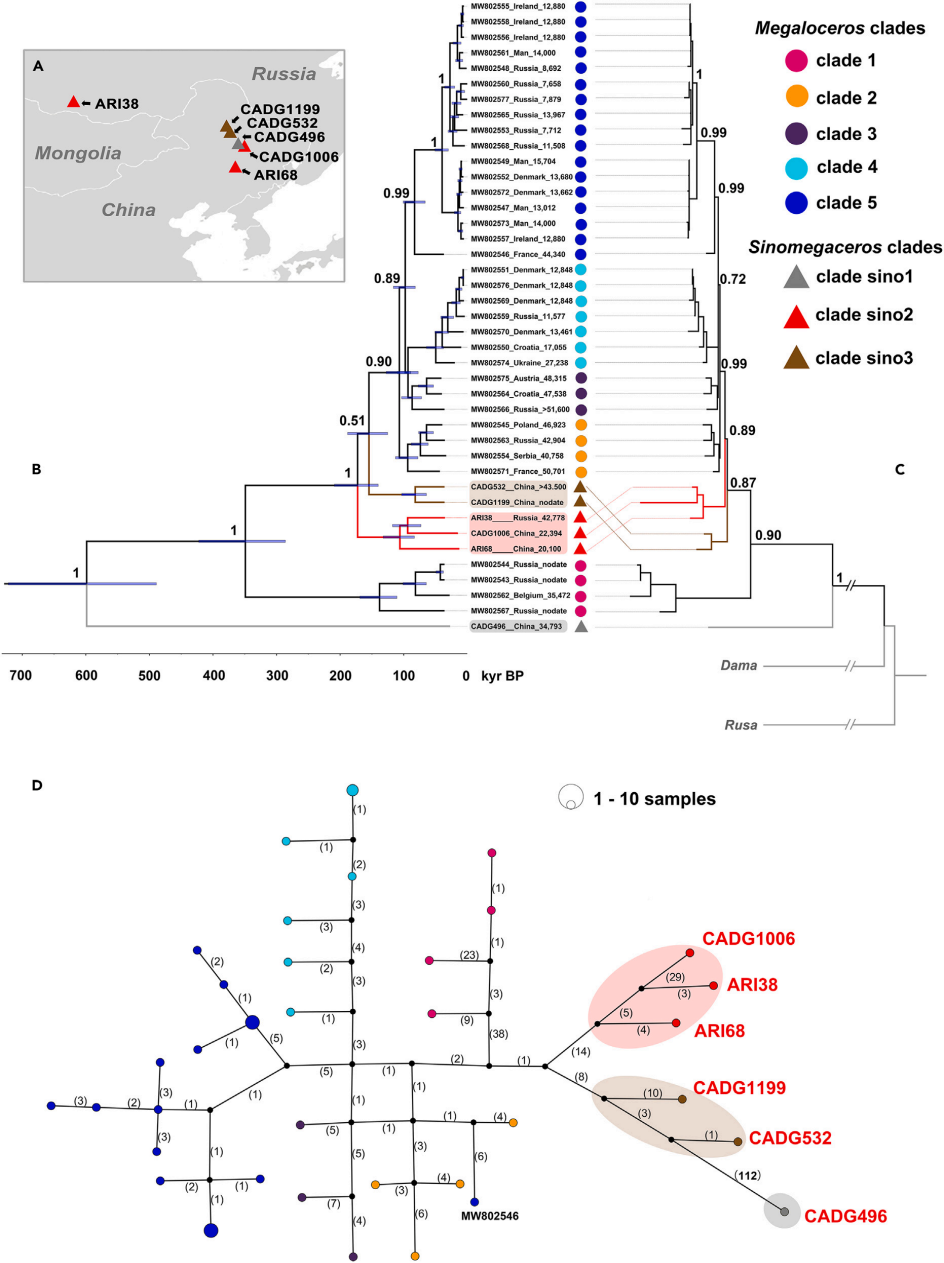
Geographical locations of the six ancient DNA containing samples and the maternal phylogeny of giant deer (A) Geographical locations of our six samples that yielded aDNA in this study. (B) MCC tree of all giant deer in BEAST based on 14,670 bp homologous mitogenome sequences. For each node, Bayesian posterior probabilities are shown at the branches. Blue node bars show 95% HPD of the divergence times. The locations and dating information are shown after the accession numbers or sample names. Our samples are marked with different colored triangles indicating clades sino1–3. The samples from publications are shown with different colored circles to indicate different clades referring to Rey-Iglesia et al.^2^ (C) ML tree of 14,670 bp homologous mitogenome sequences of giant deer with *Dama* and *Rusa* serving as outgroup. Bootstrapping was performed with 1,000 replicates. The bootstrapping support values of each node are shown near the nodes. (D) Median-joining network of 41 giant deer individuals to show 36 haplotypes based on 327 mutation sites calculated with PopART. Black circles represent missing haplotypes. The numbers represent mutational steps between haplotypes. Haplotypes are colored corresponding to MCC tree and ML tree. 35 *Megaloceros* individuals were divided into 30 haplotypes, while six *Sinomegaceros* formed six haplotypes, respectively. The number with bold font indicates the significant far distance (112 mutation steps) of the haplotype represented by CADG496 to its nearest haplotype. The haplotypes’ detailed information and Median-joining network based on regions can be found in Figure S3.

With addition of our *Sinomegaceros* individuals (Figure 3A), the phylogenetic topology is almost consistent between the maximum clade credibility (MCC) tree (Figure 3B) and the maximum-likelihood (ML) tree (Figure 3C). Additional to the five clades (clades 1–5) of *M. giganteus* revealed by Rey-Iglesia et al.,^2^ the *Sinomegaceros* individuals did not cluster together within one mitochondrial clade but formed three separate clades (clades sino1-3 in Figures 3B and 3C). Clade sino1 represents an earlier divergent branch than previous clade 1. Clade sino2 and sino3 were placed between clade 1 and clades 2–5 of *Megaloceros*, with either clade sino2 or clade sino3 sitting in the root position of clades 2–5 and the others, in the MCC tree and ML tree, respectively. In the MCC tree that shows timescales of divergences, the split event for clade sino1 occurred at approximately 599 ka (95% HPD: 489–722 ka). After the divergence of clade 1 at 349 ka (95% HPD: 286–422 ka), clade sino2 and sino3 diverged at approximately 173 (95% HPD: 140–209 ka) and 155 ka (95% HPD: 125–188 ka), respectively. As further divergence events occurred, clade 3, clade 4, and clade 5 appeared between approximately 107 and 97 ka, consistent with Rey-Iglesia et al.^2^

Our median-joining network analyses (Figures 3D and S4) detected 327 mutation sites from the 14,670 bp homologous mitogenome of 41 giant deer individuals and divided them into 36 haplotypes, including 30 haplotypes for 35 *Megaloceros* and six haplotypes for six *Sinomegaceros*. Regarding the geographical distribution of specimens, all *Megaloceros* haplotypes show no distinct geographical pattern, while for *Sinomegaceros*, except for a Siberian specimen ARI38, five specimens all originate from northeastern China. It is worth mentioning that the haplotype represented by CADG496 has a significantly long mutation distance (112 mutation sites, marked with bold font in Figure 3D), relative to the distances between other haplotypes, even further than that between *Sinomegaceros* and *Megaloceros*. The haplotype represented by MW802546, a *Megaloceros* specimen from France that has been assigned to clade 5 in both the previous study^2^ and our phylogenetic trees (Figures 3B and 3C), shows a relatively high mutation distance from other haplotypes of clade 5.

The average nucleotide diversity (Pi) and the slide window analysis of the Pi results show that *Sinomegaceros* has the highest Pi value, while the European population of *Megaloceros* has the lowest Pi value (Table 2; Figure S4). Tajima’s D of *Sinomegaceros* and *Megaloceros* was calculated as −0.94905 and −0.11504, respectively. Considering the geographical distributions of *Megaloceros*, European population has a positive Tajima’s D of 0.08502, while Ural and Siberian populations have a negative Tajima’s D of −0.37357.

**Table 2:** Average Pi and Tajima’s D of *Sinomegaceros* and *Megaloceros*

Population	Number of specimen (random sampling)	Average Pi	Average Tajima’s D
*Sinomegaceros*	6	0.00883	−0.94905
*Megaloceros*	6 of 35 (30 times)	0.00365	−0.11504
*Megaloceros* (Ural and Siberia)	6 of 23 (30 times)	0.00579	0.08502
*Megaloceros* (Europe)	6 of 12 (30 times)	0.00268	−0.37357

### Population dynamics and isotope analysis

**Figure 4:**
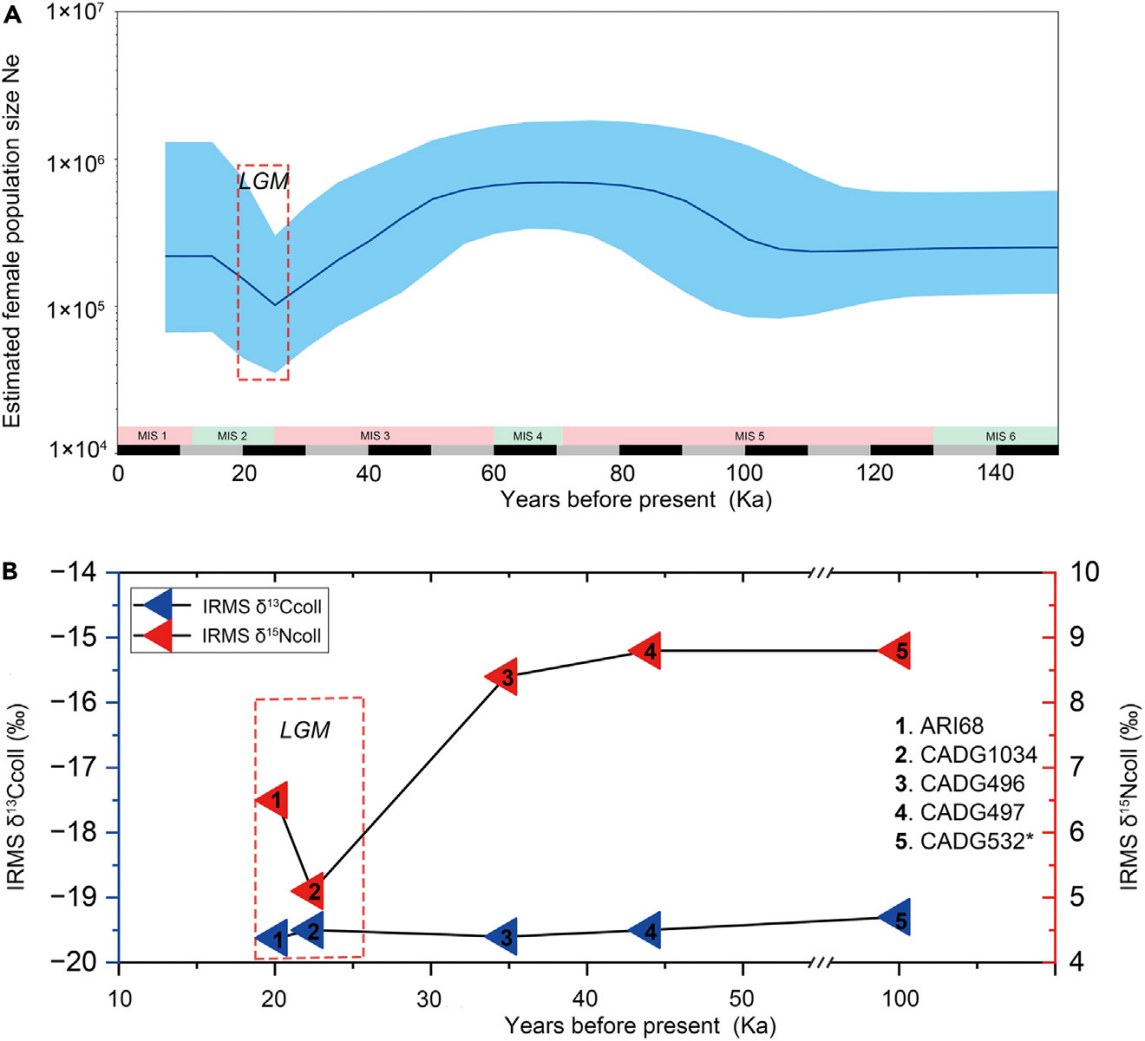
Population dynamics and stable isotope analyses of giant deer (A) Bayesian skyline plot based on 14,670 bp homologous mitogenome sequences of 35 *Megaloceros* samples from previous publications and six newly obtained *Sinomegaceros* individuals. The x axis is years before present (ka); the y axis stands for the estimated effective female population size (*Ne*). Dark blue line represents median value, and blue area is the 95% HPD limits. Pink and cyan shading represent MIS stages, and the dotted box stands for the LGM. (B) Trend of stable isotope values of five *Sinomegaceros* individuals (CADG496, CADG497, CADG532, CADG1006, and ARI68) from northeastern China with radiocarbon dating information. Blue and red triangles represent δ^13^Ccoll and δ^15^Ncoll values of specimens, respectively. Five specimens were marked with numbers 1–5. ∗The median age of CADG532 is a molecular age estimated using BEAST-1.10.4^44^ (Figure S2).

The Bayesian skyline plot (Figure 4A) shows that the effective population size of giant deer started to increase from the second half of MIS 5. After a stable period in MIS 4 and the onset of MIS 3, its population shrank until about 24 ka in the LGM. Then, there was a brief recovery in population size through 24–13 ka. In the Holocene, it kept stable until its extinction (∼8 ka) in Central Asia. In Figure 4B, the δ^13^Ccoll values remain stable, while δ^15^Ncoll values show a significant decline during the LGM.

## Discussion

In this study, we present the first mitogenomic investigation of the eastern *Sinomegaceros*, including specimens of *S. pachyosteus* and *S. ordosianus*, and combine them with published mitogenomes from *M. giganteus* that have been divided into five clades.^2^ In the phylogenetic trees, one *Sinomegaceros* clade (clade sino1), represented by *S. pachyosteus* CADG496, is in the basal position of all giant deer in the mitochondrial tree. Two other *Sinomegaceros* clades are placed paraphyletically between *M. giganteus* clades 1 and 2, the topology of the MCC and ML trees slightly differing in terms of which sino clade settles in the basal position of clades 2–5 and the others. Therefore, neither *M. giganteus* nor *Sinomegaceros* is monophyletic in the mitogenome trees. The median-joining network shows the same clustering pattern as the trees, with the haplotype represented by CADG496 having significantly more mutation sites and longer variation distance from both *Megaloceros* and other *Sinomegaceros* haplotypes. This implies that a clear boundary at the mitogenome level seemingly did not exist between *Sinomegaceros* and *Megaloceros*.

However, mitochondrial data represent a maternally inherited single locus and does not reflect the full evolutionary history of populations or species. The same scenarios of interlaced mitochondrial lineages between two groups have also been found in other mammals. For example, the extinct Eurasian cave hyena was found to be intermixed with the extant African spotted hyena (*Crocuta crocuta*) in the mitochondrial phylogeny.^25^^,^^27^^,^^28^ The straight-tusked elephant (*Palaeoloxodon* spp.) was detected to fall within the mitogenetic diversity of extant forest elephants (*Loxodonta cyclotis*).^29^^,^^30^ As revealed by nuclear analyses in these two mammalian groups,^31^^,^^32^ recovery of nuclear sequences from eastern and western giant deer would allow us to determine if the mitochondrial phylogeny indicates either gene flow between the two *Sinomegaceros* species and between the two giant deer genera, or incomplete lineage sorting,^33^ i.e., that several different mitogenomic lineages that diverged long before became fixed in different ancestral populations. If gene flow between the two giant deer genera proves to be correct, it would suggest that the conclusion of van der Made and Tong^6^ based on morphological evidence, that there was no evidence of interaction between western and eastern giant deer since their divergence, may need revision. Moreover, the position of clade sino1 (CADG496) might be the “correct” position for all *Sinomegaceros*, in the case that clades sino2 and sino3 had picked up genetic material from *Megaloceros* clades 2–5. It is too soon to consider any revisions to the current specific or generic taxonomy of these deer on the basis of our results.

Rey-Iglesia et al. concluded that two of five clades (clades 4 and 5) of *M. giganteus* recolonized central and northern Europe from southern refugia after the LGM.^2^ This conclusion has been supported by the Tajima’s D value of European *Megaloceros* population (−0.37357, see Table 2) in our study that shows a recent dispersal after bottleneck.^34^ In this study, we found representatives of a *Sinomegaceros* clade surviving during the LGM in northeastern China (Figures 3A–3C), since this clade (clade sino2) includes one specimen (ARI38) dated pre-LGM and two specimens (CADG1006 and ARI68) dated to the LGM (Table 1 and S2). Both of the other two newly detected clades contain only pre-LGM individuals, with one individual in clade sino1 AMS-dated to 34,793 cal BP and two in clade sino3 molecularly dated to 102 ka (95% HPD: 79–134 ka, CADG532) and 58 ka (95% HPD: 43–75 ka, CADG1199). Therefore, it is currently unknown whether these two clades survived later. The existence of megafaunal remains during the LGM is often a sign of refugia.^16^^,^^17^^,^^21^^,^^35^ Considering the Tajima’s D value of *Sinomegaceros* (−0.94905), it is likely that East Asian *Sinomegaceros* experienced a recolonization like European *Megaloceros*. The *Sinomegaceros* population has the highest genetic diversity, and we suppose that the combined results in our study, i.e., the early-divergent pre-LGM clade (clade sino1), the existence of pre-LGM individuals in all eastern Asian clades, and clade sino2 that contains LGM Chinese specimens, indicate that northeastern China could have been an LGM refugium for giant deer.

A remarkable result of our study concerns the specimen from the Upper Paleolithic site of Kamenka, Buryatiya, in the Transbaikal area of Siberia near Ulan-Ude.^36^ This specimen (ARI38, see Figure S1), originally identified as *M. giganteus*, groups closely with the individual of *S. ordosianus* (ARI68) from Yushu (Jilin), northeast China (Figures 3A and 3B). The sampled specimen (ARI38), a partial humerus, was found in the same assemblage as a megacerine antler base (K-93A, see Figure S5), and the radiocarbon dates of the two specimens are close.^21^ The humerus shows several morphological features in common with *M. giganteus* compared to other deer genera,^5^ confirming its megacerine identity. The antler base displays an extremely wide base of the dorsoventrally flattened brow tine, with the width of 71 mm. This is consistent with *S. ordosianus* and not with Late Pleistocene *M. giganteus* (cf. Lister^37^).^6^ DNA content of the antler was unfortunately insufficient, but the humerus (ARI38) DNA together with the antler (K-93A) morphology indicate an extension of the range of *Sinomegaceros* (pink shaded area in Figure 1), likely across Mongolia, to the Baikal region, close to (or possibly overlapping with) the known range of *M. giganteus* (black dotted line circled area in Figure 1). Other specimens from Russia (in particular those in *M. giganteus* clade 1, placed between the *Sinomegaceros* clades in the tree) are from western Siberia, group with a western European individual (from Belgium), and are referable to *M. giganteus*.

Our Bayesian skyline plot shows a continuous decline in giant deer maternal population size in MIS 3, followed by a recovery in MIS 2 (Figure 4A), which suggests a dramatic effect of climate change on population dynamics of giant deer. In Europe, Siberia, and Japan, changes in vegetation and habitat caused by climate fluctuations are considered to be the main reasons for regional extirpations of giant deer.^2^^,^^14^^,^^21^^,^^22^^,^^38^ Palynological studies have shown that the area of forest (mostly boreal coniferous forest) in northeastern China increased dramatically through 40 to 22 ka,^39^ at the expense of open or semi-open habitats. This vegetational transition may also explain the decline of δ^15^Ncoll values in Figure 4B, since the δ^15^Ncoll values are positively related to the proportion of open area.^40^ Meanwhile, paleoenvironmental studies show lake expansion, a sign of relatively warm and humid climate, in northeastern China during the period 30 to 22 ka, followed by a lake retreat reflecting dry and cold climate after 22 ka.^41^ Consistent with this evidence, the fossil record of both the *Mammuthus-Coelodonta* fauna and humans in northeastern China show dramatic increase in density during the first half of the LGM (26–22 Ka), decreasing significantly during the coldest period from 22 to 18 ka.^42^ To sum up, northeastern China experienced climatic and vegetational changes before and after 22 ka and an extremely dry and cold climate in the second half of the LGM (22–18 ka). These changes may have severely reduced megafauna population sizes, including that of giant deer. Considering the arrival of humans in northern East Asia before the LGM,^43^ possible anthropic contributions to the extirpation of giant deer in this region remain unclear and would require archaeological evidence.

### Conclusions

The newly obtained six near-complete mitochondrial genomes of *S. pachyosteus* and *S. ordosianus* correspond to three mitochondrial clades, one of which represents the earliest divergent clade in all giant deer and the other two contained within the mitogenetic diversity of *M. giganteus*. Besides the two known clades of *M. giganteus* that survived the LGM, we find a clade of *Sinomegaceros* during the LGM, which implies that northeastern China could have been a refugium for eastern giant deer. Combined with stable isotope evidence, paleoenvironmental studies, and faunal fossil records, our estimation of the population sizes suggests that climate-induced vegetation shifts may have contributed to population decline of giant deer in northeastern China.

### Limitations of the study

The failure to retrieve ancient DNA from specimens collected in Japan limited our ability to incorporate mitogenomes of giant deer across all of East Asia. Further sampling and successful obtaining of ancient DNA and radiocarbon dates from one of the three identified clades of *Sinomegaceros* that contain only one specimen will tell us if this clade may also survive the LGM in northeastern China. Nuclear genome data will detect the reason of the interlaced mitochondrial lineages of two morphologically distinct groups and further elucidate the evolutionary history of the giant deer.

## STAR★Methods

### Key resources table

**Table:** 

REAGENT or RESOURCE	SOURCE	IDENTIFIER
**Chemicals, peptides, and recombinant proteins**		

0.5 M EDTA (pH = 8)	Biosharp	Cat#BL518A
Ultra pure water	HyClone	Cat#SH30538.02
Proteinase K	Merck	Cat#1245680100
MinElute PCR Purification Kit	Qiagen	Cat#28006
NEB buffer 2	New England Biolabs	Cat#B7002S
BSA	New England Biolabs	Cat#C2312
Quick Ligase buffer	New England Biolabs	Cat#B2200S
Isothermal buffer	New England Biolabs	Cat#B0537S
Q5 High-Fidelity DNA Polymerase	New England Biolabs	Cat#M0491V
D1000 screen tape	Agilent	Cat#5067-5582
dsDNA HS assay kit	Thermofisher	Cat#Q32851
AmpliTaq Gold DNA polymerase	Invitrogen	Cat#N8080240
DNeasy Blood & Tissue Kit	Qiagen	Cat#69504
Hieff Canace Plus High-Fidelity DNA Polymerase	Yeasen	Cat#10153ES60
KAPA HiFi uracil+ premix	KAPA Biosystems	Cat#KK2801

**Deposited data**		

New sequences of *Sinomegaceros*	This study	OR263892-6, OR506264
Additional supplemental material	This study	

**Oligonucleotides**		

P5 indexing primer: AATGATACGGCGACCACCG AGATCTACAC*nnnnnnnn*ACACTCT TTCCCTACACGACGCTCTT	Gansauge and Meyer^45^	Sangon Biotech
P7 indexing primer: CAAGCAGAAGACGGCATAC GAGAT*nnnnnnnn*GTGACTGGAGTTCAGACGTGT	Gansauge and Meyer^45^	Sangon Biotech
Long range primer CEP1_Fwd: TAGCAATTATTCTACTATCCGTCCTC	Rey-Iglesia et al.^46^	Sangon Biotech
Long range primer CEP1_Rev: GAATTAGTAGGTGTCCTGCAGTAATGTTAG	Rey-Iglesia et al.^46^	Sangon Biotech
Long range primer CEP2_Fwd: TTATAGGCCTTCCACTAGCTACTCTC	Rey-Iglesia et al.^46^	Sangon Biotech
Long range primer CEP2_Rev: GTGATTGTGACTAGGAAAGAGAGGAAATAC	Rey-Iglesia et al.^46^	Sangon Biotech
Long range primer CEP3_Fwd: CTCTAATATACCCCTAATAGGCCTTG	Rey-Iglesia et al.^46^	Sangon Biotech
Long range primer CEP3_Rev: CTGAAGATGGCGGTATATAGACTGTATTAG	Rey-Iglesia et al.^46^	Sangon Biotech
Long range primer CEP4_Fwd: AAGTTAATAAGACTAAGAGGAGCTG	Rey-Iglesia et al.^46^	Sangon Biotech
Long range primer CEP4_Rev: GTGGATAGAACAACTATTGTAGGTAGAAGG	Rey-Iglesia et al.^46^	Sangon Biotech

**Software and algorithms**		

fastp-0.22.0	Chen et al.^47^	https://github.com/OpenGene/fastp
bwa-0.7.15	Li & Durbin^48^	https://github.com/lh3/bwa
SAMtools-1.3.1	Li et al.^49^	https://github.com/samtools/samtools
ANGSD-0.916	Korneliussen et al.^50^	https://github.com/ANGSD/angsd
Qualimap-2.2.1	Okonechnikov et al.^51^	http://qualimap.conesalab.org/
mapDamage2.0	Jonsson et al.^52^	http://ginolhac.github.io/mapDamage
IQtree-1.6.12	Nguyen et al.^53^	http://www.iqtree.org/
Kglign	Lassmann T.^54^	https://github.com/timolassmann/kalign
Gblock-0.91b	Talavera & Castresana^55^	https://github.com/atmaivancevic/Gblocks
BEAST-1.10.4	Suchard et al.^44^	https://github.com/beast-dev/beast-mcmc/releases/tag/v1.10.4
jModelTest-2.1.9	Darriba et al.^56^	https://github.com/ddarriba/jmodeltest2
Tracer-1.6	Rambaut & Drummond^57^	https://github.com/beast-dev/tracer
TreeAnnotator-1.8.2	Rambaut & Drummond^58^	https://github.com/beast-dev/beast-mcmc/releases/tag/v1.10.4
FigTree-1.4.3	Rambaut et al.^59^	http://tree.bio.ed.ac.uk/software/Figtree/
DnaSP-6.12.03	Rozas et al.^60^	http://www.ub.es/dnasp
PopART-1.7	Leigh & Bryant.^61^	http://www.ub.es/dnasp
Origin 2023b	OriginLab	https://www.originlab.com/

### Resource availability

#### Lead contact

Further information and requests should be directed to and will be fulfilled by the lead contact, Guilian Sheng (glsheng@cug.edu.cn)

#### Materials availability

This study did not generate new unique reagents.

#### Data and code availability

The five ancient mitochondrial sequences newly generated in this study are available at GenBank under accession numbers OR263892-6, OR506264 and is publicly available as of the date of publication.

This paper does not report original code.

Any additional information required to reanalyze the data reported in this paper is available from the lead contact upon request.

### Experimental model and study participant details

We collected 16 Late Pleistocene giant deer specimens from East Asia, including Russia, northern and eastern China, and Japan (Figures 1 and S1). Of these, two Japanese samples were morphologically identified as *S. yabei*, six samples from China and Russia as *S. ordosianus*, and six samples from China as *S. pachyosteus*. The three species can be separated on their skull, antler and mandibular morphology, supplemented by their known geographical and stratigraphic ranges.^3^^,^^5^^,^^6^ *S. yabei* is known only from the Middle to Late Pleistocene of Japan, and has a reindeer-like triangular brow tine as well as a posterior (back) tine at the base of the antler beam. *S. pachyosteus* and *S. ordosianus* are known from Middle and Late Pleistocene deposits in China, respectively. *S. pachyosteus* has a short facial region, enormous brow tine, and extreme mandibular thickening (pachyostosis) compared to the other species*. S. ordosianus* has a sagitally-oriented brow tine palmation and a downwardly- and backwardly-bent antler beam with a relatively small distal palmation. Only two of our samples (CADG1006 & CADG1199) did not show enough morphological characters for identification with certainty. Radiocarbon dating of specimens by accelerator mass spectrometry (AMS) was carried out at BETA Analytic, US and University of Oxford, UK. All dates were calibrated using the IntCal20 curve.^62^ Detailed information on these samples is provided in Table S2.

### Method details

#### Ancient DNA extraction, amplification and sequencing

The whole experimental process was completed in dedicated ancient DNA laboratory facilities at China University of Geosciences (Wuhan) (CUG), the Natural History Museum (UK), and the Natural History Museum of Denmark. Approximately 60–120 mg of bone powder for each specimen was mixed with extraction buffer consisting of 3 mL EDTA (0.5 M, pH = 8) and 0.045 mL Proteinase K (20 mg/mL) in a 15 mL centrifuge tube. After overnight incubation in a rotating hybridization at 37°C, the mixture was centrifuged for 10 min at 7,000 rpm. The supernatant was transferred into an ultrafiltration centrifugal tube (10 kDa, Millipore) and centrifuged at 7,000 rpm until 100–150 μL left. The purification step was carried out using MinElute PCR Purification Kit (Qiagen), and finally 50 μL ancient DNA extract was obtained.

Multiple DNA double-stranded libraries was constructed for each specimen using 20 μL extract of each sample following the protocol.^63^ After blunt-end repairing, adapter ligation, and fill-in steps, indexing PCR amplifications were performed using Q5 High-Fidelity DNA Polymerase (New England Biolabs) and dual primers (P5 and P7 for Illumina sequencing platform) under the following conditions: 120 s at 95°C and 17 cycles of 15 s denaturation at 95°C, 30 s annealing at 60°C and 30 s elongation at 68°C. Libraries concentration and fragments size distribution were quantified using Qubit 4.0 (Thermo Fisher Scientific) and 4145 TapeStation (Agilent Technologies). Next-generation sequencing was conducted on Illumina HiseqX platform in Annoroad Gene Technology Co., Ltd, Beijing, China.

For the samples processed at the Natural History Museum (UK) and the Natural History Museum of Denmark (ARI38, ARI68, ARI136 and ARI136), DNA extracts were USER treated prior to library build. Library build steps were the same as above, except for the index PCR, which was performed using AmpliTaq Gold DNA polymerase (Invitrogen). These libraries were sequenced on an Illumina HiSeq 2000 platform 80 bp single read at The National High-throughput DNA Sequencing Center, University of Copenhagen, Denmark.

#### Mitochondrial genome hybridization capture and sequencing

For the samples that yielded aDNA, hybridization capture was carried out (Table S2). All the capture experiments were processed in a modern molecular biology laboratory at CUG. The modern DNA extraction of red deer muscle for making bait was carried out using DNeasy Blood & Tissue Kit (Qiagen) following the instruction. Four paired primers (Table S3) were used for long-range PCR.^64^ The bait-making and mitochondrial genome hybridization capture steps followed Meyer and Kircher^63^ and Fortes and Paijmans^65^ with the minor modification that the AmpliTaq Gold Polymerase (Thermo Fisher Scientific) for post-capture amplification was replaced by Hieff Canace Plus High-Fidelity DNA Polymerase (Yeasen). The enriched libraries were purified with MinElute PCR Purification Kit (Qiagen) and then sequenced on Illumina HisqX platform in Annoroad Gene Technology Co., Ltd, Beijing, China.

For the samples processed at the Natural History Museum and the Natural History Museum of Denmark, hybridization capture was carried out using a Mybaits (MYcroarray) custom-design giant deer mitogenome array based on the consensus sequences from Immel et al.^11^ The standard MYbaits v.3.0 protocol was applied with hybridization for 30 h at 55 °C at all relevant steps. Post-capture amplification was performed using KAPA HiFi uracil+ premix (KAPA Biosystems) for 14 cycles following MYbaits v.3.0 protocol set up recommendations. Captured libraries were pooled equimolar and sequenced on an HiSeq 2000 80 bp single read.

### Quantification and statistical analysis

#### Data processing

Sequencing raw reads were trimmed with fastp-0.22.0^47^ and reads less than 30 bp were discarded. The trimmed reads were mapped against a complete mitochondrial genome of *M. giganteus* (GenBank: MW802558) using bwa-0.7.15 ‘aln’^48^ with default options expect for disabled seed. SAMtools-1.3.1^49^ was used to sort mapped reads and remove duplicates with options ‘sort’ and ‘rmdup’. The final mitochondrial consensus sequence was produced using ‘-doFasta 2’ in ANGSD-0.938,^50^ setting a minimum base depth of 2 (-setMinDepthInd 2) to avoid DNA damage and sequencing errors. Calculation of reads coverage and analyses of DNA damage were processed using Qualimap-2.2.1^51^ and mapDamage2.0^52^ with default parameters, respectively (Figures S6 and S7).

#### Phylogenetic analysis

To investigate the phylogenetic position of the eastern giant deer individuals, we carried out ML phylogenetic analysis with IQtree-1.6.12.^53^ The six newly obtained mitochondrial genomes were aligned with 35 ancient sequences of *M. giganteus*, four modern sequences of *Dama* and *Rusa* serving as outgroup (Table S4) using the Kglign^54^ online offered by EMBL-EBI.^66^ Finally, 14,670 bp homologous sequences were obtained using Gblock-0.91b^55^ with default parameters for subsequent analyses.

To estimate the divergence time of different giant deer groups, we carried out BEAST analysis based on 14,670 bp homologous mitogenomes consisting of our six newly obtained sequences and 35 *M giganteus* sequences from previous publications^2^^,^^11^ using BEAST-1.10.4.^44^ We determined the best suitable evolutionary model of ‘HKY+I+G’ for the mitogenome dataset using jModelTest-2.1.9.^56^ The mean value of clock rate was set to 1.65 × 10^−8^ substitutions/site/year with a standard deviation of 0.01 based on Artiodactyla studies.^2^^,^^67^ The ages of samples with accurate date were used to calibrate the evolutionary rate as prior information in the tip-dating sets. The Markov chain Monte Carlo (MCMC) was run for 80 million generations, sampling every 1,000 generations. Tracer-1.6^57^ was used for checking the outfiles of BEAST analysis to ensure that all effective sample sizes (ESS) values are over 200. We finally constructed the MCC tree with TreeAnnotator-1.10.4^58^ and modified it using FigTree-1.4.3.^59^

The maternal demographic history was constructed based on the same homologous mitogenomes of 41 giant deer using BEAST-1.10.4.^44^ We set a same clock rate of 1.65 × 10^−8^ substitutions/site/year and evolutionary model of ‘HKY+I+G’. Bayesian skyline plot was visualized with Tracer-1.5.^57^

For CADG532, the specimen dated beyond the limitation of radiocarbon dating, and CADG1199, the one we did not radiocarbon date, we carried out an estimation with BEAST-1.10.4.^44^ All giant deer specimens with definite ages were used as tip-date calibrating to estimate the median age of these two specimens. The other parameters are set as above.

The median-joining network was constructed in PopART-1.7^61^ based on all 327 mutation sites from 14,670 bp homologous mitogenomes obtained from 41 giant deer individuals using DnaSP 6.^60^ First, we constructed a median-joining network corresponding to MCC and ML clades. Then, we divided all giant deer specimens geographically into three regions: Europe, Ural&Siberia, and China, and constructed the second median-joining network. Meanwhile, we calculated sliding window nucleotide diversity (Pi) of *Sinomegaceros* (N = 6), *Megaloceros* (N = 35), *Megaloceros* (Europe, N = 23), and *Megaloceros* (Ural&Siberia, N = 12) with length of 100 bp and step of 25 bp using DnaSP 6, respectively. Considering that our dataset contains only 6 samples, we calculated nucleotide diversity and Tajima’s D by randomly sampling 6 samples from each of the other three datasets with 30 sampling times, and then calculated the average nucleotide diversity and Tajima’s D.

#### Stable isotopes

To evaluate the stable isotope signal of our ancient giant deer from northeastern China, the stable isotope (carbon (^13^C) and nitrogen (^15^N)) values of five giant deer (ARI68, CADG496, CADG497, CADG532, and CADG1006) that have been radiocarbon dated were measured with collagen by BETA Analytic, US and University of Oxford, UK. With specimen weights of about 0.5–1.0 g each, the above professional laboratories performed all measurement operations in accordance with operating specifications (ISO/IEC 17025:2017). Combined with the ages of these specimens, we visualized the trends of two stable isotopes over time using Origin 2023b.

## Supplemental information

Document S1. Figures S1–S7

Table S1. Samples' detailed information. All personal collection specimens can be accessed via lead contact, Guilian Sheng (glsheng@cug.edu.cn), related to Figure 1&3 and STAR Methods

Table S2. Rawdata mapping statistics of giant deer (reference sequence: M. giganteus, MW802558), related to STAR Methods

Table S3. Primer pairs used for generation of the mitochondrial baits for capture referred to sequence AB245427 (Cervus elaphus), related to STAR Methods

Table S4. Published and new sequences for phylogenetic analyses in this study, related to STAR Methods
