# Supplementary material for: Relationships of Late Pleistocene giant deer as revealed by Sinomegaceros mitogenomes from East Asia
Source: iScience. 2023 Nov 7;26(12):108406. doi: 10.1016/j.isci.2023.108406 (PMC10690636; doi:10.1016/j.isci.2023.108406)
Supplement: Document S1. Figures S1–S7 [file mmc1.pdf]

iScience, Volume *26*

Supplemental information

Relationships of Late Pleistocene giant deer as revealed by *Sinomegaceros* mitogenomes from East Asia

Bo Xiao, Alba Rey-lglesia, Junxia Yuan, Jiaming Hu, Shiwen Song, Yamei Hou, Xi Chen, Mietje Germonpré, Lei Bao, Siren Wang, Taogetongqimuge, Lbova Liudmila Valentinovna, Adrian M. Lister, Xulong Lai, and Guilian Sheng

Supplementary Materials:

Figure S1. Pictures and collection information of 16 new giant deer individuals in this study, related to Discussion and STAR Methods.

Figure S2. The results of molecular dating for CADG532 (a) and CADG1199 (b), related to Results and STAR Methods.

Figure S3. Median-joining network of 36 giant deer haplotypes based on 327 mutation sites calculated with PopART. Black circles represent missing haplotypes. The numbers represent mutational steps between haplotypes. All specimens are geographically divided into three groups (Europe, Ural&Siberia, and China), related to Figure 3.

Figure S4. Sliding windows analysis of nucleotide diversity using 327 mutation sites of 41 giant deer. Tests run four times for *Sinomegaceros*, *Megaloceros*, *Megaloceros* (Europe), and *Megaloceros* (Ural&Siberia), respectively. The blue line shows the nucleotide diversity of the whole mitogenome. Population, numbers of specimens, Pi and Tajima's D are shown in blue font, related to Table 2.

Figure S5. Antler base cf. *S. ordosianus* from Kamenka, Russia, no. K-93A, showing very wide, flat brow tine characteristic of this species. (a) Dorsal view; (b) anterior view. Photos Mietje Germonpré,

related to Discussion.

Figure S6. Cytosine deamination frequency inferred from Late Pleistocene giant deer samples analyzed in this study. ARI38 and ARI68 were treated with USER enzyme (NEB) in DNA extraction, thus they show a no-damage pattern compared to other specimens (CADG496, CADG532, CADG1006, and CADG1199) without USER treatment, related to STAR Methods.

Figure S7. Mapped single-end read length distribution of ancient individuals, related to STAR Methods.

Table S1. Samples' detailed information. All personal collection specimens can be accessed via lead contact, Guilian Sheng (glsheng@cug.edu.cn). Related to Figure 1&3 and STAR Methods.

Table S2. Rawdata mapping statistics of giant deer (reference sequence: *M. giganteus*, MW802558), related to STAR Methods.

Table S3. Primer pairs used for generation of the mitochondrial baits for capture refered to sequence AB245427 (*Cervus elaphus*), related to STAR Methods.

Table S4. Published and new sequences for phylogenetic analyses in this study, related to STAR Methods.

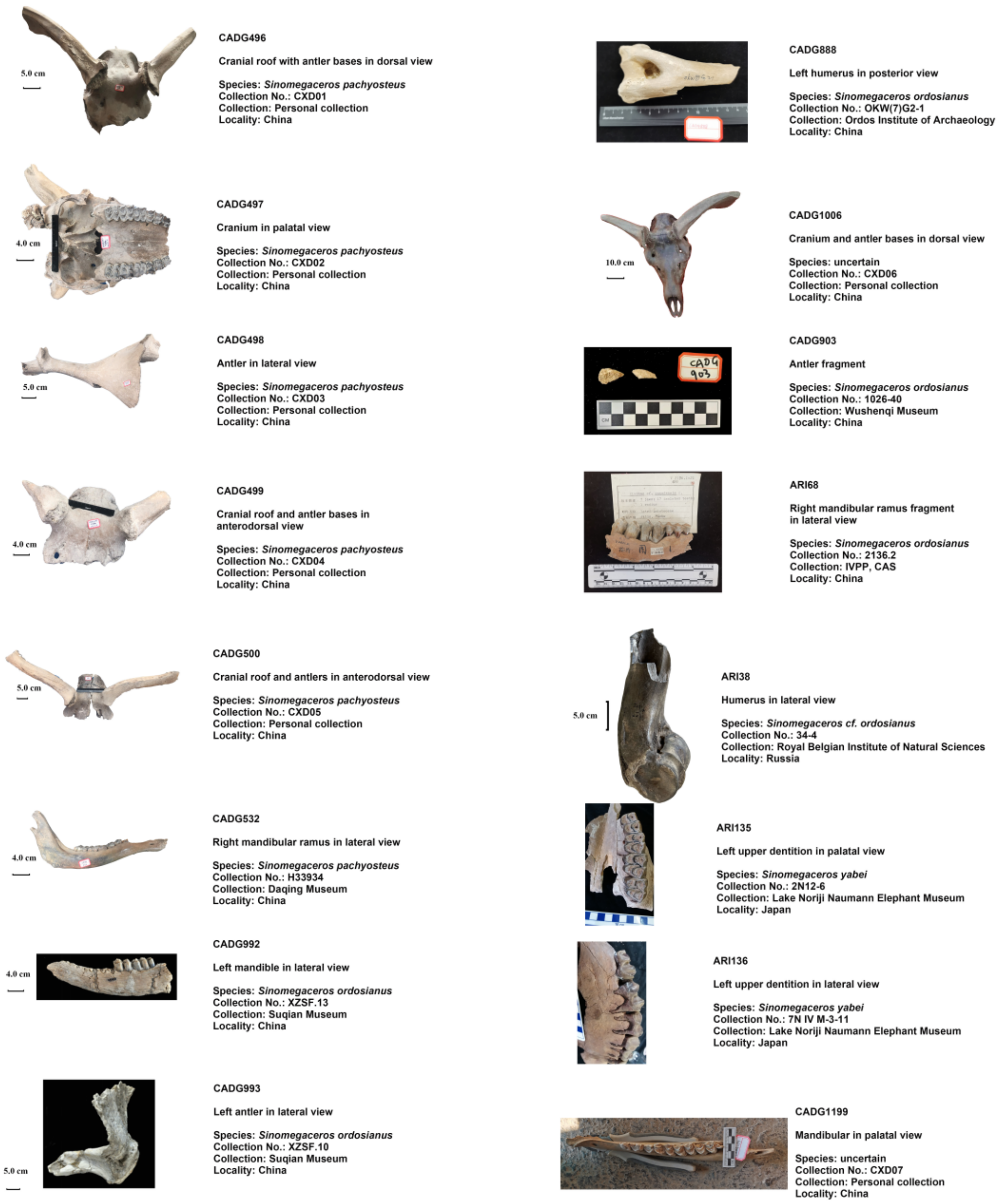

Figure S1. Pictures and collection information of 16 new giant deer individuals in this study, related to Discussion and STAR Methods.

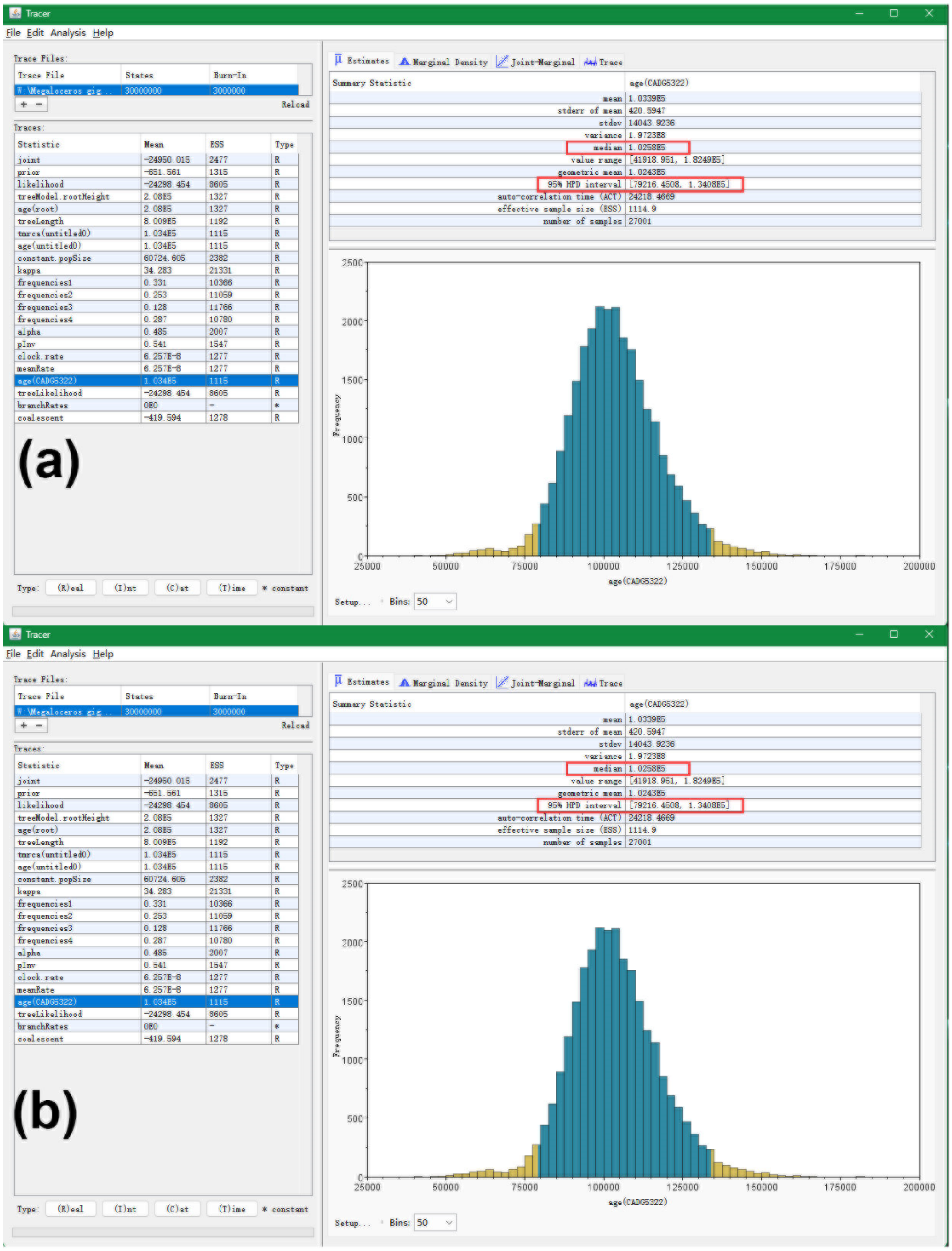

Figure S2. The results of molecular dating for CADG532 (a) and CADG1199 (b), related to Results and STAR Methods.

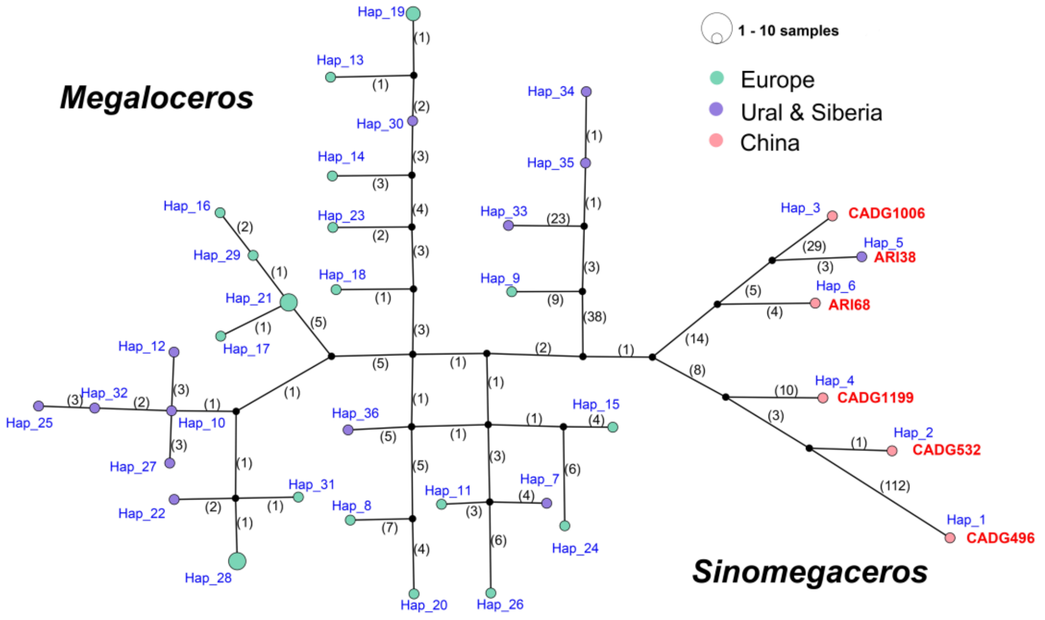

Hap_1: CADG496
Hap_2: CADG532
Hap_3: CADG1006
Hap_4: CADG1199
Hap_5: ARI38
Hap_6: ARI68
Hap_7: MW802563
Hap_8: MW802564
Hap_9: MW802562
Hap_10: MW802565
Hap_11: MW802545
Hap_12: MW802568
Hap_13: MW802569
Hap_14: MW802570
Hap_15: MW802571
Hap_16: MW802573
Hap_17: MW802572
Hap_18: MW802574
Hap_19: MW802576 MW802551
Hap_20: MW802575
Hap_21: MW802547 MW802549 MW802552
Hap_22: MW802548
Hap_23: MW802550
Hap_24: MW802546
Hap_25: MW802577
Hap_26: MW802554
Hap_27: MW802553
Hap_28: MW802556 MW802555 MW802558
Hap_29: MW802557
Hap_30: MW802559
Hap_31: MW802561
Hap_32: MW802560
Hap_33: MW802567
Hap_34: MW802544
Hap_35: MW802543
Hap_36: MW802566

Figure S3. Median-joining network of 36 giant deer haplotypes based on 327 mutation sites calculated with PopART. Black circles represent missing haplotypes. The numbers represent mutational steps between haplotypes. All specimens are geographically divided into three groups (Europe, Ural&Siberia, and China), related to Figure 3.

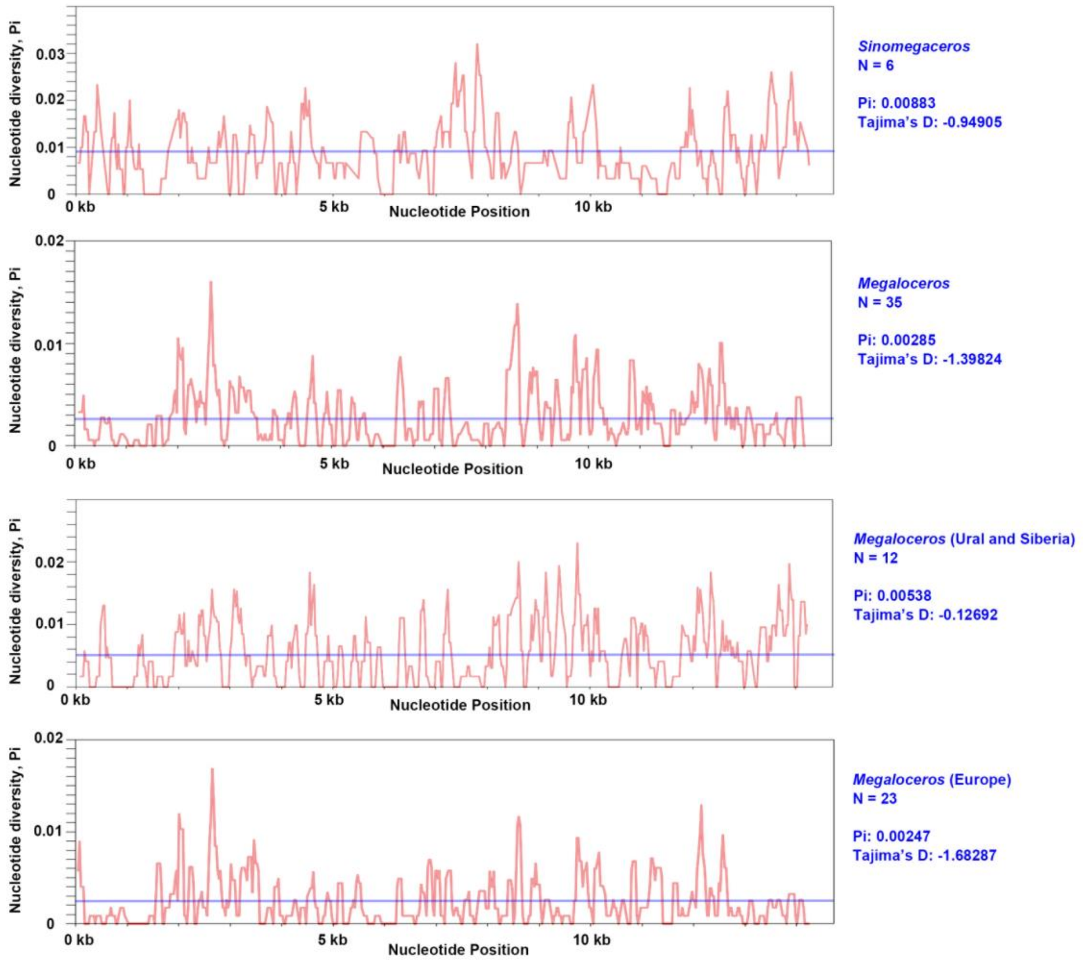

Figure S4. Sliding windows analysis of nucleotide diversity using 327 mutation sites of 41 giant deer. Tests run four times for *Sinomegaceros*, *Megaloceros*, *Megaloceros* (Europe), and *Megaloceros* (Ural&Siberia), respectively. The blue line shows the nucleotide diversity of the whole mitogenome. Population, numbers of specimens, Pi and Tajima's D are shown in blue font, related to Table 2.

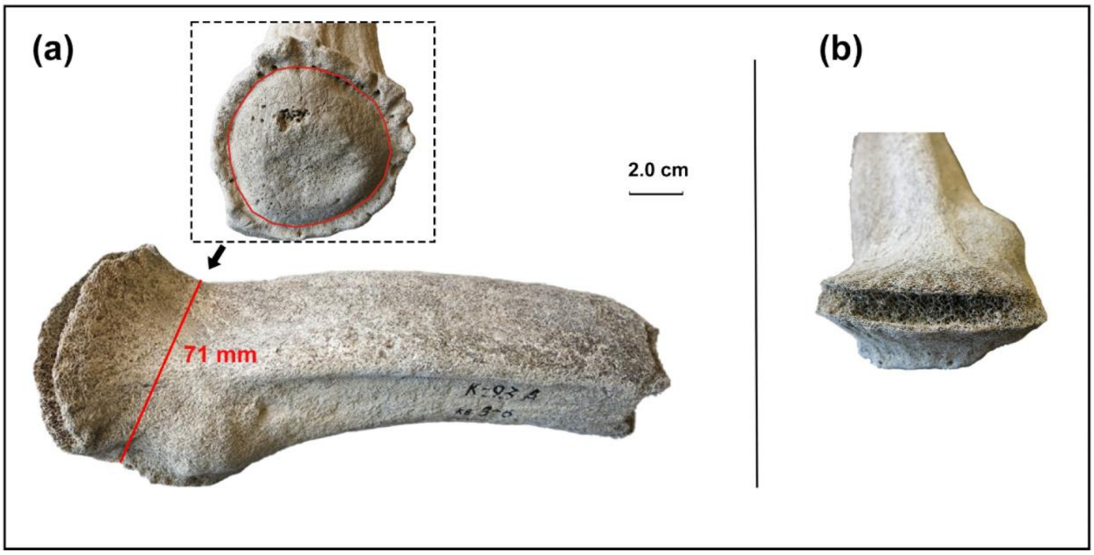

Figure S5. Antler base cf. *S. ordosianus* from Kamenka, Russia, no. K-93A, showing very wide, flat brow tine characteristic of this species. (a) Dorsal view; (b) anterior view. Photos Mietje Germonpré, related to Discussion.

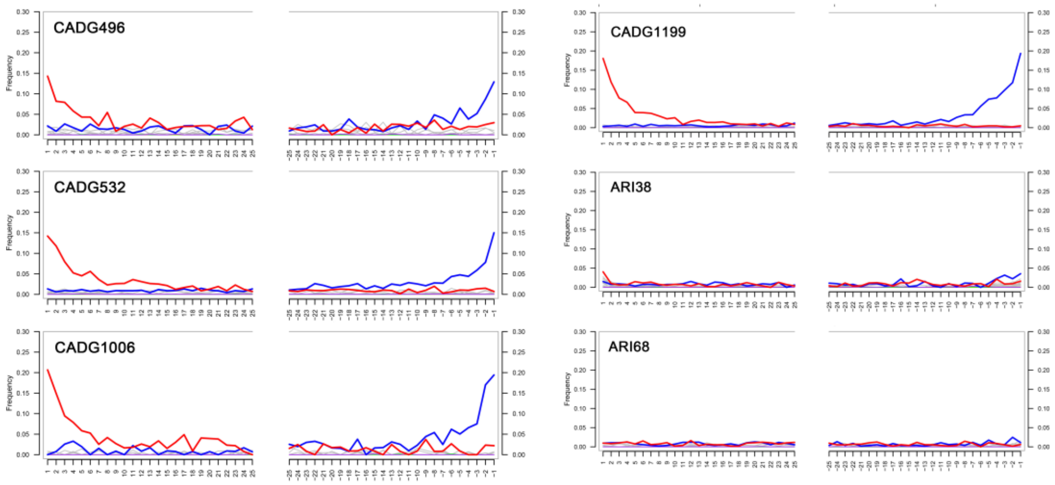

Figure S6. Cytosine deamination frequency inferred from Late Pleistocene giant deer samples analyzed in this study. ARI38 and ARI68 were treated with USER enzyme (NEB) in DNA extraction, thus they show a no-damage pattern compared to other specimens

(CADG496, CADG532, CADG1006, and CADG1199) without USER treatment, related to STAR Methods.

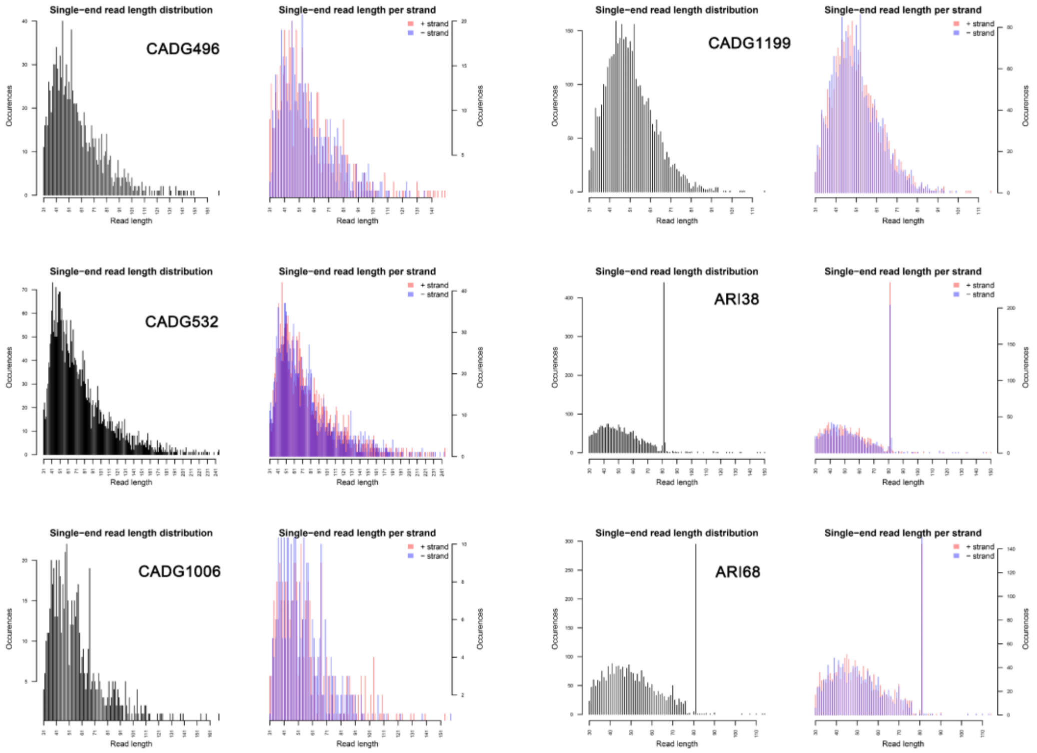

Figure S7. Mapped single-end read length distribution of ancient individuals, related to STAR Methods.
